# Initiatives on early detection and intervention to proactively identify health and social problems in older people: experiences from the Netherlands

**DOI:** 10.1186/s12877-015-0131-z

**Published:** 2015-10-30

**Authors:** Manon Lette, Caroline A. Baan, Matthijs van den Berg, Simone R. de Bruin

**Affiliations:** Centre for Nutrition, Prevention and Health Services, National Institute of Public Health and the Environment, Bilthoven, The Netherlands; Scientific Centre for Transformation in Care and Welfare (Tranzo), University of Tilburg, Tilburg, The Netherlands

**Keywords:** Early detection, Preventive home visit, Health and social problems, Older people, Frailty, Qualitative research

## Abstract

**Background:**

Over the last years, several initiatives on early detection and intervention have been put in place to proactively identify health and social problems in (frail) older people. An overview of the initiatives currently available in the Netherlands is lacking, and it is unknown whether they meet the preferences and needs of older people. Therefore, the objectives of this study were threefold: 1. To identify initiatives on early detection and intervention for older people in the Netherlands and compare their characteristics; 2. To explore the experiences of professionals with these initiatives; and 3. To explore to what extent existing initiatives meet the preferences and needs of older people.

**Methods:**

We performed a qualitative descriptive study in which we conducted semi-structured interviews with seventeen experts in preventive elderly care and three group interviews with volunteer elderly advisors. Data were analysed using the framework analysis method.

**Results:**

We identified eight categories of initiatives based on the setting (e.g. general practitioner practice, hospital, municipality) in which they were offered. Initiatives differed in their aims and target groups. The utilization of peers to identify problems and risks, as was done by some initiatives, was seen as a strength. Difficulties were experienced with identifying the target group that would benefit from proactive delivery of care and support most, and with addressing prevalent issues among older people (e.g. psychosocial issues, self-reliance issues).

**Conclusion:**

Although there is a broad array of initiatives available, there is a discrepancy between supply and demand. Current initiatives insufficiently address needs of (frail) older people. More insight is needed in “what should be done by whom, for which target group and at what moment”, in order to improve current practice in preventive elderly care.

## Background

Due to population ageing, health systems face the challenge to offer care and support to an increasing number of older people. Therefore, governments stimulate older people to participate in society and to live at home for as long as possible, with support of formal and informal caregivers [1–5]. Many people age in good health and remain active participants in society throughout their lives. Still, the prevalence of frailty, (multi)morbidity and disability increases with age. Frailty, (multi)morbidity and disability often lead to restrictions in social participation, reduced self-reliance and care dependence, which in turn may lead to the utilization of long-term care and support services [6–9].

It is believed that early detection of risks and early intervention can delay or even reduce frailty and disability [10–13]. Therefore, over the last years, several countries (e.g. United Kingdom, USA, Canada, Australia, Denmark, Japan) have experimented with initiatives that aim to proactively identify and address health and social problems in older people [14–19]. These initiatives have been described in literature under a variety of names, such as ‘preventive home visits’, ‘geriatric care management’, ‘identification of frailty in primary care’, and ‘population-based multidimensional geriatric assessment’ [14, 16, 17, 19–21]. In this paper, we refer to these initiatives with the term ‘initiatives on early detection and intervention’.

Also in the Netherlands, initiatives on early detection and intervention are taking place. Although some of these initiatives have been described in literature [9, 21–24], at present no comparisons have been made between the different types of initiatives. It is therefore unknown whether these initiatives overlap or complement each other. Moreover, it has not been investigated whether existing initiatives meet the preferences and needs of older people. More insight into this matter is desirable, particularly because, despite developments regarding the delivery of more person-centred care [25], older people do not always feel that the health services provided to them meet their needs and preferences [26, 27]. As a result, issues important for older people are often insufficiently addressed [26].

Therefore, the aims of this qualitative study were threefold: 1. To identify initiatives on early detection and intervention for older people in the Netherlands and compare their characteristics; 2. To explore the experiences of professionals with these initiatives; and 3. To explore to what extent existing initiatives meet the preferences and needs of older people. In this study, older people were defined as people aged 65 or older.

Interest in what constitutes best practice in (preventive) elderly care is growing [28], particularly among municipalities. In the Netherlands, for instance, under the Public Health Act [in Dutch: Wpg], introduced in 2008, municipalities became amongst others responsible for the implementation of preventive services for older people, such as early detection and intervention. Additionally, as in many countries [29–31], also in the Netherlands reforms in the healthcare system recently took place, resulting in the shift of responsibilities for health and social care services from the national government to municipalities [32, 33]. Due to this shift, municipalities have become responsible for supporting vulnerable citizens (e.g. frail older people, informal caregivers) to participate in society and live at home for as long as possible. This is for instance done by offering services to them that support self-efficacy and social interactions or offer them respite (e.g. adult day care services, transport facilities, domestic aid, adapted housing). Municipalities are free to set their own policy with regard to these new responsibilities, which is why there is an increasing need for information to support policy development with regard to (preventive) elderly care [33, 34], and thus for information on how to organize early detection and intervention.

## Methods

### Study design and participants

This qualitative explorative descriptive study was performed between September 2013 and January 2014. Semi-structured interviews were conducted with a purposeful sample of experts in preventive elderly care and older people in order to gather in-depth information about (experiences with) initiatives. Because many initiatives in the Netherlands have not yet been described in literature, we consulted grey literature and databases and websites from Dutch research and knowledge institutes [35–38] as a preparation for the interviews. This provided a preliminary overview of the different initiatives on early detection and intervention in the Netherlands. Based on expert information obtained during the interviews and by snowballing, this preliminary overview was complemented and adjusted.

### Experts

We conducted 12 interviews with 17 experts who are renowned for their expertise in research, policy and/or practice with regard to preventive elderly care. We interviewed four researchers, six policymakers, two care professionals, two managers of social care organisations and three persons who were both researcher and care professional. The experts had specific knowledge on one or more (categories of) initiatives as well as a broad perspective on preventive elderly care in general. Initial selection of experts took place based on our preliminary literature search which provided us insight into the experts involved in the initiatives. Further selection took place by snowballing until we had included multiple experts from various professions and initiatives. Experts were contacted via email and asked for their cooperation. Seven experts were interviewed individually and five interviews were attended by two experts. Ten interviews were conducted face-to-face, and two interviews were conducted over the phone. Interviews with experts took an average of one hour to make sure all interview topics could be discussed in-depth.

### Older people

We conducted three group interviews with a total of 21 volunteer elderly advisors (VEAs). The total group consisted of 11 men and 10 women whose age ranged from 57 to 78 years. Their mean age was 69 years. VEAs are trained volunteers, most of whom are 65 years or older, who visit older people at their homes. The aim of their visits is to help older people with their problems, amongst others by providing advice and practical support (for more information, see Table 1). The reason we interviews VEAs was twofold: 1) they were approached as experts on the category ‘home visits by VEAs’, and 2) they were approached as older people. Since this study was an explorative study, and considered as a first step to better understand older people’s needs and preferences with regard to early detection and intervention, we asked VEAs to speak both on behalf of themselves as older people and as proxies for the frail older people they visit and as such to function as advocates for the frail older people they visit. We put an invitation letter in the newsletter of the umbrella organisation for Dutch elderly organisations in order to recruit VEAs. Further enrolment took place through the VEAs that responded to the newsletter invitation. To take diversity into account, group interviews took place in the east, centre and north-west of the Netherlands. All group interviews were conducted by pairs of researchers. Group interviews took 1,5 h on average to make sure all interview topics could be discussed in-depth.

### Ethics

This study does not fall under the scope of the Dutch Medical Research Involving Human Subjects Act (in Dutch; WMO) and therefore did not need to undergo a review by a Medical Ethical Committee. At the start of the interview, we explained the purpose of the interview, how we would handle the respondents’ data and how their confidentiality would be maintained. We verified whether they understood their involvement. All interviews were audiotaped with permission of the respondents and transcribed verbatim.

### Interview topics

Experts: the focus of the interviews with experts was twofold:

1. Characteristics of particular (categories of) initiatives on early detection and intervention in the Netherlands. The interviews covered the following topics:
  Goal, setting, target population, initiator
  Methods used in the initiative to identify frail older people or older people at risk of frailty, and methods used to assess problems and risks. Since there is no consensus on the definition of frailty and its determinants [39], we adopted a broad interpretation. Initiatives were included in our study, regardless of how “frailty” was defined in the initiative
  Scope (i.e. health, wellbeing, participation, living circumstances etc.)
  Effectiveness of the initiative
  Follow-up of the initiative (such as preventive programmes, care plans and case management)

These topics provided descriptive information on particular (categories of) initiatives on early detection and intervention, and were used to verify and complete information previously obtained through grey literature.

2. Overall experiences and views on early detection and intervention. The interviews covered the following topics:
  Experienced alignment between initiatives on early detection and intervention
  Overall strengths and weaknesses of initiatives on early detection and intervention
  Possibilities for improvement of existing initiatives

Older people: interviews with VEAs focused on needs and preferences with regard to early detection and intervention. The interviews covered the following topics:

- Potential personal experiences with early detection and intervention
- Views (their own and by proxy of the people they visit) on early detection and intervention
- Preferences with regard to early detection and intervention (e.g. setting, kind of professional, scope and approach)

### Data analysis

For data-analysis, the framework analysis method was used [40–42]. The code structure, or analytical framework, was developed based on the principles of both and deductive and an inductive approach [42]. This implies that predetermined codes, derived from the topic list for the interviews, were used for the development of the initial framework (i.e. deductive approach). By reading several interview transcripts and establishing the relevance and coherence of recurring themes, additional codes were added to the analytical framework (i.e. inductive approach). When no new concepts emerged from reviewing successive data, the analytical framework was finalized and used to assign codes to relevant passages of the interview transcripts [41, 42]. Two researchers (ML and SdB) coded the interview transcripts and checked the others’ coded transcripts. We organized a consensus meeting between ML and SdB to discuss differences and to reach consensus for all codes. A computer program for qualitative data analysis (ATLAS.ti 7.1.3) was used to aid in the analysis of the coded transcripts by sorting data according to themes. Findings were discussed between all authors and draft study findings were shared with a varied group of respondents to validate findings through ‘member checking’ [43]. The respondents confirmed our results and/or provided valuable comments which helped us to further hone our findings.

## Results

### General characteristics of categories of initiatives on early detection and intervention in the Netherlands

#### Goals

We identified a wide variety of initiatives on early detection and intervention, aimed at both older people in general and frail older people specifically. Based on their aims, the initiatives were clustered in two groups: 1. Initiatives that aimed to detect older people at risk of deterioration in order to provide a preventive follow-up programme (see group 1 in Table 1) and 2. Initiatives that aimed to detect problems (and needs) with regard to health and wellbeing in frail older people in order to optimize (current) delivery of health and social care (see group 2 in Table 1).

Within these two groups, the initiatives were further categorized based on the setting in which they were provided and their scope, which resulted in a total of eight categories of initiatives. Group 1 was divided into two categories ((i) initiatives by Elderly Health Centres and (ii) informative home visits by volunteers) and group 2 into six categories ((iii) initiatives by general practitioner (GP) practices, (iv) initiatives in hospitals and during hospital transfers, (v) initiatives by health and social care professionals who visit older people at their homes, (vi) initiatives by community nurses/an integrated neighbourhood approach, (vii) home visits by VEAs and (viii) home visits by municipalities).

With these categories, we aim to provide an overview of what types of initiatives are currently offered in the Netherlands and to enable comparison between them. It should, however, be noted that within these categories, variation exists between individual initiatives with regard to various characteristics (e.g. target population, initiator, screening instruments, scale), according to the local context in which the individual initiative is offered. For example, initiatives by GP practices (category 3) took place in at least 170 GP practices across the country for which varying definitions of frailty, designs and target populations were used, whereas the initiative by health and social care professionals who visit older people at their homes (category 5) was only implemented in one area in the south-west of the Netherlands.

#### Setting, target population, and initiator

The setting of initiatives varied from GP practices (category 1 and 3), hospitals (category 4), home care organisations (categories 1, 5 and 6), social care organisations (category 2), volunteer organisations (category 7) and municipalities (category 8). The target groups were older people in general (categories 1, 2 and 6), older people at risk of frailty (categories 3, 4 and 5) and frail older people (categories 7 and 8). Initiatives in categories 1 to 6 were initiated by health or social care professionals or volunteers. Initiatives in category 7 and 8 were initiated by older persons themselves or their social network by contacting a VEA (category 7) or the municipality (category 8).

#### Instruments and scope

Five out of eight categories of initiatives used a screening instrument or questionnaire such as the Groningen Frailty Indicator (GFI), the Identifications of Seniors at Risk-Hospitalized patients (ISAR-HP), EASY-Care Two-step Older persons Screening (EASY-Care TOS) and Intermed (categories 1–4 and 8) to identify frailty and/or problems and needs of older people. Three categories of initiatives did not use a screening instrument (categories 5–7). Initiatives focused on different domains, which varied from physical, social and mental functioning (initiatives 1, 3–6) to wellbeing, safety, living circumstances, self-reliance and social participation (initiatives 2, 4, 6–8). Initiatives on early detection and intervention provided by health care professionals mainly addressed physical health and health problems, such as diabetes, cardiovascular diseases and overweight. The majority of the initiatives also focused on psychological health and considered mental problems and risks to be of importance.

#### Cost-effectiveness

With regard to (cost-)effectiveness of initiatives, not much information was available. Currently, several initiatives are being evaluated in the Netherlands. Preliminary results are inconsistent and difficult to compare due to variation in focus and design of initiatives. However, according to GPs, screening for frailty and setting out goals and agreements in a care plan structured their daily practice of elderly care. Furthermore, trained geriatric care professionals collaborated more with other professionals and were more aware of locally available care and support services for older people than professionals who were not specially trained in geriatric care. The (cost-)effectiveness of initiatives to prevent health and social problems provided by volunteers have hardly ever been evaluated.

#### Follow-up

The follow-up as described here refers to the types of interventions that were initiated based on the potential problems that were detected. The follow-up of initiatives in category 1 and 2 mainly consisted of the provision of advice and information and referral to other health and social care professionals if that was deemed necessary. Follow-up of initiatives in categories 3–8 merely focused on the improvement of current delivery of care and support by for instance designing personal care plans, delivering coordinated care or providing practical support.

### Overall experiences and views of experts on early detection and intervention

When analysing the data on experts’ experiences and views on early detection and intervention, five themes emerged that provide recurrent and unifying ideas regarding the initiatives in general. These themes are as follows: (i) definition of frailty and identification of frail older people, (ii) scope of initiatives, (iii) alignment of initiatives, (iv) effectiveness of initiatives, and (v) follow-up to early detection. The themes will be described below, illustrated with representative quotes from the interviews.

#### Definition of frailty and identification of frail older people

Respondents indicated that difficulties exist with regard to identifying those older people who would benefit from early detection and intervention most. There is no consensus on the definition of frailty and its determinants and hence nor on the target population or on the most effective method or screening instrument to identify the target groups. As a result, target groups varied widely across initiatives as well as the methods to identify frail older people or older people with specific problems and risks with regard to health and wellbeing.

The following quotation illustrates the experienced difficulties regarding defining the target population:

*From the start, we were in two minds about this project. In retrospect, it’s always more clear than during the project itself. So, looking back at what we wrote in the project proposal, it’s already there. It says: we aim to focus on frail older people, so older people living in disadvantaged neighborhoods and the oldest old with co-morbidity; but those two groups are completely different!* (Researcher 1)

#### Scope of initiatives

Even though the majority of the initiatives considered psychological health to be of importance, several experts indicated that problems in these domains were hardly ever addressed in practice and that problems such as loneliness and depression often went unnoticed. Respondents indicated to struggle with bringing up this topic or to give the topic little priority during their limited time with older people. VEAs stated that trust in professionals is very important for older people to be willing to discuss issues related to psychological health. Time, interest and an open conversation between the professional and the older person are factors that can enhance this trust, while the utilization of screening instruments or questionnaires may discourage older people to bring up psychological issues. As a community nurse put it:

*Such a screening list is fine, but the way I see it, it also depends on the trust you put in people and the relationships you can build. That is, that you make the effort to get a full picture of a person’s situation. (Community Nurse 1)*

Therefore, it was suggested by several respondents involved in social care or volunteer organisations that community nurses and volunteers (such as VEAs) might be in a better position to detect issues related to psychological health and loneliness than GP’s or an elderly health centre, as they foster trust and confidence and tend to have more time to discuss potential issues, since they make home visits and they hardly ever use screening instruments. As a VEA stated:

*And when we come in, then you have a totally different atmosphere. Then you get those problems out on the table. (VEA 1)*

#### Alignment of initiatives

Since older people use a variety of health and social care services, they can be exposed to early detection and intervention in different settings, and as such by different types of initiatives. Respondents indicated that there is often little collaboration between health care professionals, social care professionals and volunteers involved in the different types of initiatives. Being often unaware of each others’ activities, this results in inefficiency and unnecessary overlap of preventive activities and other (health) services.

This lack of alignment between different health and social care services is illustrated by the following quotation:

*There are so many agencies willing to support older people: Humanitas [social services and community building organisation], the Salvation Army, De Wering [organisation of social workers], community centres, senior citizens’ associations, residential homes with their own volunteers. This makes it very difficult for older people to know where to ask for help. […] There are so many people who believe ‘older people are lonely, we need to do something about that’, and start with another service. And I think, ‘there are so many services already. What about integrating all of them, before starting up something new’.* (VEA 2)

In the interviews, three factors were mentioned that hamper the collaboration between different disciplines. First, initiatives tend to adopt a single disciplinary approach. Health care professionals mainly detect health problems and risks whereas social care professionals mainly detect problems related to wellbeing. Care professionals from different disciplines are little apt to integrate their services, which leads to fragmentation. Second, according to VEAs, collaboration between professionals and volunteers is also quite an issue as professionals consider volunteers their competitors, making them reluctant to share responsibilities with volunteers. Third, care professionals, researchers and policy makers indicated that the current financing structure of elderly care in the Netherlands impedes collaboration between disciplines or integration of different initiatives. Preventive care, curative care and long-term and social care are funded from different resources in the Netherlands.

The following quotation reflects the challenges regarding cooperation between different health and social services:

*There are always two sides to this kind of things. One, regarding cooperation, it is not always easy for disciplines that have to work together, to look past the end of their noses. Two, daily organizational hassles: how do you manage to meet? The fact that one discipline is being paid for attending meetings while another is not, doesn’t make it any easier. That is not conducive to getting things done. (Researcher 2)*

#### Effectiveness of the initiatives

Researchers indicated that initiatives that target a more select group of older people (e.g. people over 65 years old at high risk of loss of functioning during hospitalisation) could be more effective than initiatives that target a more generic population of older people. They also indicated that many instruments for the identification of frail older people might not be sufficiently sensitive to identify those older people who would benefit most from the initiatives and proactive care and support. However, the weak evidence base of many initiatives was also pointed out as a problem. As a researcher and policy-maker put it:

*What you want is evidence, good evidence. Five years ago we started these projects because our hearts told us: this should be the right type of care. If you’d ask me now, ‘is this good care?’, then I’d say ‘yes, it is’. After seeing the evidence, whether I need to adjust my ideal, I do not know. Everyone is still very much preoccupied with their ideals; ideologically, it should be like this. (Researcher 3)*

*Within healthcare, but also in the social domain, you must know what interventions are effective. With heart surgery, you know: it works or it does not. But in the social domain to achieve that goal is tricky, assuming you have a clear, well formulated goal and you also know: I will achieve this goal because I’m doing this and that. This causality is also a difficult issue. (Policymaker 1)*

With regard to initiatives provided by volunteers, experiences of VEAs and managers of welfare organisations were positive and they suggested possible benefits of initiatives provided by volunteers compared to initiatives provided by professionals. Characteristics of these initiatives that may positively influence effectiveness are for example that volunteers such as VEAs performed home visits only at the explicit request of older persons themselves or people in their social network. Furthermore, volunteers tend to be more approachable, to have a more practical approach than professionals do and to better empathize with older people with regard to their preferences and needs.

The following quotation reflects the positive experience with older people as volunteers:

*A grey head of hair, that appeals to them. And that’s why our motto is: for and by older people. It works, and not always bring in a professional. (Policymaker 2)*

#### Follow-up to early detection

According to several professionals, follow-up of the detected problems and risks is rarely properly considered. For initiatives aiming to detect older people at risk of deterioration in order to provide a preventive follow-up programme, experts indicated that effective follow-up programmes are often lacking. Effective follow-up programmes for older people exist for a selected number of problems and risks only, including high blood pressure, smoking, exercise, loneliness and depression. The following quotation shows the implications of this:

*Screening for something for which there is no effective intervention makes no sense, and unfortunately, that’s true for almost everything. There are very few exceptions. (Researcher 3)*

Furthermore, researchers indicated that access to follow-up programmes is often poor in terms of timing and location. They are only offered a few times a year or outside the neighbourhood.

For initiatives aiming at detecting problems and needs with regard to health and wellbeing in frail older people in order to optimize current delivery of health and social care, experts also indicated that a consistent overview of effective follow-up is lacking.

### Experiences and views of older people on early detection and intervention

When analysing the data on older people’s experiences and views on early detection and intervention, three themes emerged that provide recurrent and unifying ideas regarding the initiatives in general. These themes are as follows: (i) approach, (ii) scope of initiatives, and (iii) setting of initiatives.

**Table 1:** Overview of initiatives on early detection and intervention for older people in the Netherlands

Category (based on setting)	Target population	Goal	Initiator	Health/social care professionals involved	Scope	Follow-up	Screening methods	Scale
*Group 1: initiatives aiming to detect older people at risk of deterioration in order to provide preventive interventions* ^*a*^								
1. Initiatives by Elderly Health Centres [63–66]	Varies per initiative: target populations based on for example age, SES, health insurer, location of health care use	To prevent or early detect physical and psychological problems in (frail) older people	Varies per initiative: Home care organisation, municipal mental health care organisation, GP, nurse practitioner, community nurse	Varies per initiative: Community nurse, Municipal Health Services, (occupational) physician, community psychiatric nurse	Physical, psychological and social functioning	Provision of information and advice on lifestyle, preservation of independence and control. Referral to other professionals if necessary	Varies per initiative: For example: an instrument covering 3 domains: 1. Screening for frailty (Groningen Frailty Indicator); 2. Screening for health problems (Intermed) 3. Screening for wellbeing (Groningen Wellbeing Indicator)	Various locations were identified across the Netherlands. Per elderly health centre, a varying group of people was exposed to the initiative (e.g. a whole community; only people from certain GP practices; people affiliated with a specific health insurer)
2. Informative home visit by volunteers [67–69]	All people in a municipality who are 75 years and older	To bring community services to the attention of older people and to detect unidentified problems	Volunteer from welfare/volunteer organization	Welfare/volunteer organization, professional elderly advisor	Health, wellbeing, living circumstances, social participation	Provision of advice on services that can facilitate self-reliance and participation	Screening instruments are often not used. In some municipalities, a questionnaire or list with topics regarding activities, social relations, mobility, finances, nutrition is used	Informative home visits are offered by local welfare organisations in various municipalities across the Netherlands. Within those municipalities, every person over 75 years is exposed
*Group 2: initiatives aiming to detect problems (and needs) with regard to health and wellbeing in frail older people in order to optimize (current) delivery of health and social care* ^*a*^								
3. Initiatives by GP practices [23, 24, 51, 70–74]	Older people living at home, who are at high risk of frailty. Age categories differ per initiative, e.g. people aged 65 and over; people aged 75 and over.	To identify frail older people in the population, and provide proactive care if necessary	Primary care	GP, nurse practitioner, community nurse. Other health and social care professionals if necessary, according to the problems that are identified.	Physical, psychological and social functioning	Design and execute a personal care and support plan	Varies per initiative: Screening methods for frailty, e.g. GP registries, screening instruments such as Groningen Frailty Indicator.	At least 8 types of initiatives were identified that were practiced in various locations in the Netherlands. The initiatives included approximately 170 practices and approximately 16.300 (frail) older people were exposed to the initiatives
							Screening instruments for problems and risks related to health, wellbeing and living circumstances, e.g. Resident Assessment Instrument, EASY-Care TOS	
4. Initiatives in hospitals and during hospital transfers [22, 75, 76]	Patients over 65 who are at high risk for loss of function during hospitalisation	To prevent loss of function during and after hospitalisation	(Research) nurse in hospital	Geriatric nurse, transfer nurse, geriatrician, case manager. Other health and social care professionals if necessary, according to the problems that are identified	Preservation of functioning, self-reliance and quality of life	Delivery of proactive care during hospitalisation combined with coordinated after care after hospital discharge	Varies per initiative: ISAR-HP, VMS (for screening frailty). GAS-plan, geriatric assessment (for screening for problems and risks)	At least 2 types of initiatives were identified in hospitals in the south-west and north-west of the Netherlands. Based on these initiatives, a minimum of 500 older people were exposed to the initiatives
5. Initiative by health and social care professionals who visit older people at their homes [77]	Older people living at home who are frail or at risk of frailty	To early detect psychosocial problems and risks	Health and social care professionals who visit older people at their homes, e.g. nurse from a home care organization, VEA, community nurse	Professionals from municipal (mental) health care organization, nurse from a home care organization, VEA, community nurse	Psychosocial problems and risks (e.g. loneliness, depression, alcoholism, elderly abuse)	Referral to the required services for their psychosocial issues	Screening instruments are used by some professionals (e.g. the Geriatric Depression Scale, GDS). Professionals mostly use their “gut-feeling”	This initiative was identified in one area in the south-west of the Netherlands
6. Initiatives by community nurses/integrated neighbourhood approach [78–80]	Varies per initiative: the community in general. Some of the initiatives are targeted at frail older people	To gain insight into the problems and needs in a community and facilitate people to keep control over their own lives	Community nurse (sometimes in combination with other professionals that are active in the community, e.g. social workers, district policemen)	Various health and social care professionals, according to the problems and needs that are identified	Various domains. For older people mainly health, wellbeing, safety and living situation	Provision of information, practical support, after care; referral to other professionals; facilitation of involvement of family caregivers	In some initiatives screening instruments are used. An “open conversation” without using any instruments is often preferred.	Initiatives by community nurses are offered in various neighbourhoods across the Netherlands
7. Home visits by volunteer elderly advisors (VEA) [81, 82]	Older people needing help, who contacted the elderly organisation	To facilitate self-reliance by offering practical support and contacting professionals if necessary	Initiated by older person or someone in his social network	VEA, others professionals according to the problems and needs that are identified	Health, wellbeing, living circumstance, participation	Provision of advice and practical support	In some cases, a topic list is used, but screening instruments are mostly not used. An “open conversation” without using any instruments is preferred.	VEAs are active through elderly organisations or local welfare organisations in various municipalities across the Netherlands.
8. Home visits by municipalities (so-called “kitchen-table conversations”). [83]	Older people living at home who requested support from municipal services and facilities	To evaluate the extent to which older people are self-reliant and able to participate in society, the support they receive from their social network, and the care and support that would be necessary from the municipality. All intended to facilitate self-reliance and social participation.	Initiated by older person, possibly in consultation with professional	Several possibilities, but always commissioned by municipality: social care consultant of the municipality, employee from (social) welfare organization, client support organization, health care organization.	All life domains (e.g. living, working, income/debt, education, health, lifestyle, leisure activities, social activity, mobility, practical skills).	Provision of support from municipal social services (e.g. home care, adult day care services) if older people are not self-reliant or able to independently, or with the support from their social network, participate in society.	Varies per municipality, e.g. Self-reliance matrix (ZRM) and the Vitality Indicator	In principle in all municipalities in the Netherlands. However, due to large reforms of the long-term care system (that involve municipalities), not all municipalities are able to offer kitchen table conversations yet.

^a^Based on their aims, the eight categories of initiatives can roughly be divided into two groups: 1. ‘initiatives aiming to detect older people at risk of deterioration in order to provide preventive interventions’ and 2. ‘initiatives aiming to detect problems (and needs) with regard to health and wellbeing in frail older people in order to optimize (current) delivery of health and social care’. It should, however, be noted that individual initiatives from certain categories from for instance group 2 would meet the objectives of group 1 more (and the other way around). This is mainly due to local variation in how the initiatives are being practiced

#### Approach

VEAs indicated that many initiatives have little appeal to older people. They may perceive the initiatives as patronising, as these tend to take over whatever control they still have over their own lives. This is illustrated by the following quotation:

*I feel like the government thinks that all older people are unhealthy, have dementia, and I don’t know what else…That is not true. […] I think the government’s presentation of us older people is downright wrong. That’s a shame. That image has to change over there. We talk ourselves into it. (VEA 3)*

#### Scope of initiatives

VEAs indicated that problems and risks related to wellbeing, living circumstances and social participation are particularly important to older people and that professional help focusses too much on physical health. Older people prefer to get more practical support that would enhance their self-reliance, like advice and help with administrative tasks, finances or completing forms for services. Furthermore, older people would like support in preventing or decreasing loneliness, for example by getting information about opportunities for social interaction with other older people.

#### Setting of initiatives

Older people prefer to receive help and support from people they trust, who take time for them and who understand their outlook on life. Peers are considered less threatening than professionals and older people feel taken more seriously by people their own age. Home visits as a means to identify problems and risks, specifically focusing on social participation and self-reliance, were generally preferred to questionnaires and screening instruments.

Despite the fact that older people do not perceive initiatives to identify physical health problems and risks as an immediate need, VEAs indicated that if necessary, older people would prefer them to be provided by GPs. Generally, older people see the GP as an authority who regards their problems and needs objectively. This makes a GP less threatening than, for example, the municipality, whose objective is to determine whether the older person qualifies the services requested from the municipality. Therefore, older people do not necessarily perceive initiatives provided by the municipality as a means to objectively help and support them.

*What is very important, is the notion that ‘the elderly’ does not exist. Nor is there a standard solution. (VEA 4)*

## Discussion

The aim of this qualitative study was to identify existing initiatives on early detection and intervention in the Netherlands, to explore the experiences with these initiatives in the professional field and to explore whether the initiatives meet older people’s needs and preferences. To the best of our knowledge, this study is the first to make such an inventory of existing initiatives from different settings, and to determine whether supply meets demand. Although this study was performed in the Netherlands, the issues raised and the lessons learned from the experiences of experts are also considered of importance for other countries, particularly those countries that are also experimenting with preventive elderly care in order to enable older people to participate in society and live independently at home for as long as possible. This study shows the wide range of categories of initiatives that are implemented in the Netherlands, which is not entirely reflected in the literature.

The initiatives we identified are organized in various settings and focus on various domains. The large variety observed across initiatives is in line with previous studies, which showed that proactive detection of problems and risks among older people is an incoherent concept, with much variation in setting and design [17–19]. The initiatives are set up with good intentions; however it is not yet clear which initiatives are most beneficial to whom. Moreover, they often fail to meet the preferences and needs of older people. Since the effectiveness of most initiatives have not yet been evaluated, comparison with regard to effectiveness is not possible.

Several strengths and weaknesses of the different categories of initiatives were identified. In line with previous research [27, 44], home visits and a trusting relationship between the older person and professional were identified as strengths both by experts and older people. Weaker aspects of initiatives were related to the methods used to identify the target group that would benefit from these initiatives and the lack of focus on domains important for older people, such as (independent) functioning, wellbeing and participation in society. Overall, the large variation in initiatives found in this study is a strength because it allows for a broad group of older people to be reached and a broad range of domains to be covered. This is important because older people are a heterogenic group. However, the downside is that it is an obstacle to cooperation and coordination between different stakeholders and to determining what works for whom in which context.

Those initiatives that have been evaluated were mostly initiatives provided by GP’s or community/practice nurses. Evidence on effectiveness of these initiatives is, however, inconsistent. This is in line with the findings of earlier studies. Some studies found no effects of initiatives on early detection [19, 45], whereas others suggest that on certain conditions, initiatives may be effective in terms of increased functional status, reduced mortality or a reduction in nursing home admissions. Such conditions include a focus on multiple domains, multiple follow-up home visits, target groups of older people at lower mortality risk and of relatively young age [20] and a combination of interdisciplinary teamwork addressing health and social problems [18].

Furthermore, several methods to identify target groups that would benefit from prevention of health and social problems have recently been evaluated. [21, 46, 47]. Comprehensive Geriatric Assessment (CGA) seems to be effective in the hospital geriatric care setting [48, 49], but might be difficult to implement in primary care [50]. A two-step method using a short instrument or routine primary care data to make a first selection of patients who could benefit from CGA might be more efficient there [51, 52]. A comparison between several methods in primary care found that a short patient questionnaire was most accurate [47]. However, our study shows that this may not be in line with the preferences of older people.

Our finding that aspects such as (independent) functioning, wellbeing and participation in society are more important to older people than aspects related to physical health confirms findings in previous qualitative studies, of older people preferring initiatives to focus on their psychosocial context [26, 27, 44]. Phelan et al. [53] also found that for older people, successful ageing is a multidimensional concept equivalent to wellbeing. Furthermore, older people’s expectations of early detection and intervention (i.e. focus on social issues and wellbeing) are very different from their actual aims of cure and prevention (i.e. prevention of health problems and disability) [27]. This confirms our finding that the domains on which many the initiatives focus do not always meet the preferences and needs of older people.

### Methodological considerations

A strength of this study was that we interviewed a wide range of experts active in varying settings of preventive elderly care (e.g. primary care, hospitals, social care, volunteer organisations). This way, we were able to provide a comprehensive overview of initiatives, which were not primarily medically oriented. Furthermore, many experts also had extensive knowledge on preventive elderly care in general and were able to put their knowledge and experience in a wider perspective. Willingness to participate among experts was high; every expert we approached either participated or referred us to another expert better qualified with regard to the scope of our interview.

We incorporated the views of older people themselves, which allowed us to compare their needs and preferences with the available initiatives. We included older people from various regions in the Netherland and from both rural and urban areas, in order to account for different issues that arise in different regions and areas of the country (e.g. with regard to the availability of services, social cohesion in the neighbourhood). The current number of interviews allowed for data saturation. However, it should be noted that the older people that were interviewed were VEAs. Therefore, they are not representative for all older people, including frail older people. VEAs are generally more active and emancipated. However, some VEAs had experiences with some of the initiatives on early detection and intervention themselves. Furthermore, they do visit older people who are frail and less active in society, and who also have experiences with at least one initiative on early detection and intervention. During the interviews, VEAs were therefore encouraged to also speak as proxies for the older people they visited. As such, we were also able to include some of the views of more vulnerable older people. However, we should acknowledge that the answers provided by the VEAs did probably not cover issues and preferences of frail older people completely. This explorative study, however, provides some general lessons with regard to the preferences and needs of older people which may well provide a starting point for future research, in which it is recommended to study needs and preferences of older people, including those who are frail, more in-depth.

Another limitation of our study was the relatively small number of respondents and uneven distribution of number of respondents across the fields of policy, practice and research. This may have caused some bias, with some professions being overrepresented. By validating our findings from completed interviews with experts from other domains in subsequent interviews, we aimed to prevent potential personal and disciplinary bias. Furthermore, after data analysis we used ‘member checking’ to inquire whether our results were faithfully interpreted, whether they contained errors and whether they made sense to respondents from different professions.

### Implications

In the Netherlands, as in several other countries, reforms in the healthcare system are taking place. This includes decentralisation of responsibilities for health and social care services from the national government to local authorities [29–33], including the responsibility for (preventive) elderly care. This implies that municipalities have become responsible for stimulating participation and independent living of older people. Municipalities could therefore take an active role to better align existing initiatives on early detection and intervention by for instance appointing a local coordinator who ensures that the appropriate steps with regard to prevention and follow-up care are taken by the appropriate professionals. This may also enable integration of care and support provided by professionals from different disciplines. Alignment of initiatives may further be facilitated by better alignment of the different financing structures of care and support provided by the different disciplines involved in (preventive) elderly care.

As was suggested in previous research, our study underlines that in order for initiatives to better meet the preferences and needs of older people, initiatives should focus on problems with functioning and maintenance of independence and wellbeing, rather than on a specific disease and its consequences [54]. Therefore, integration of initiatives focusing on psychological health and wellbeing and initiatives focusing on physical problems is important. Furthermore, insight into the target groups for whom preventive interventions could be most beneficial is necessary. Recent research shows that preventive interventions among purposely selected older people result in more detected problems (and thus in more people in need of care and support) than interventions among randomly selected older people [55]. Older people who experience certain life transitions that are known to increase the risk of frailty and the use of care and support (e.g. moving house, becoming a widow(er), a strongly decreasing social network and hospital admissions [56–58]) may for instance benefit from early detection and intervention more than older people aged 65 years and older in general. This way, care and support services can be more tailored to their needs and resources.

This study shows that many initiatives are implemented on a larger scale without any evidence base. Existing initiatives might benefit from a critical assessment according to the criteria for responsible screening [59]. Some of these criteria are also essential for initiatives on early detection and intervention, like the need for a reliable screening method and the availability of effective follow-up interventions. Further research should address identified knowledge gaps, like the most effective method for identifying the target population and the effectiveness of initiatives that have not yet been evaluated, such as initiative provided by volunteers.

In line with Stuck et al. [20] and Beswick et al. [18], also in our study some promising aspects of initiatives for frail older people were identified, such as multiple home visits, multidimensional screening and assessment, intensive case management, providers with geriatric training and experience and referral to and coordination of community services [60]. The importance of trust and the use of peers that was highlighted in our study need further examination and it is advised to consider incorporation of these aspects in recently started initiatives. Furthermore, the trade-off between care provided by volunteers versus GPs should be further examined, as this might indicate at which point older people are open to preventive interventions by GPs. Finally, further research on how factors that limit or facilitate inter-professional teamwork should be addressed and how organizational initiatives can be used to improve health care and health outcomes for older people may help improve interdisciplinary collaboration [61, 62]. Such insights may support stakeholders and municipalities to make more evidence-based decisions with regard to the design of local strategies for prevention, care and support to the increasing number of older people.

## Conclusion

Although there is a broad array of initiatives available, there may be a discrepancy between supply and demand. There may also be a risk current initiatives insufficiently address needs of (frail) older people. More insight is needed in “what should be done by whom, for which target group and at what moment”, in order to improve current practice in preventive elderly care.

## Abbreviations

VEA: Volunteer elderly advisor
GP: General practitioner
GFI: Groningen Frailty Indicator
ISAR-HP: Identifications of Seniors at Risk-Hospitalized Patients
EASY-Care TOS: EASY-CareTwo-step Older persons Screening
